# Participatory translational science of neurodivergence: model for attention-deficit/hyperactivity disorder and autism research

**DOI:** 10.1192/bjp.2023.151

**Published:** 2024-04

**Authors:** Edmund J.S. Sonuga-Barke, Susie Chandler, Steve Lukito, Myrofora Kakoulidou, Graham Moore, Niki Cooper, Maciej Matejko, Isabel Jackson, Beta Balwani, Tiegan Boyens, Dorian Poulton, Luke Harvey-Nguyen, Sylvan Baker, Georgia Pavlopoulou

**Affiliations:** School of Academic Psychiatry, Institute of Psychiatry, Psychology & Neuroscience, King's College London, London, UK; School of Academic Psychiatry, Institute of Psychiatry, Psychology & Neuroscience, King's College London, London, UK; and Group for Research in Relationships in Neurodiversity (GRRAND), Clinical, Educational & Health Psychology, Division of Psychology & Language Sciences, Faculty of Brain Sciences, University College London, London, UK; DECIPHer, School of Social Sciences, Cardiff University, Cardiff, UK; Place2Be, London, UK; School of Academic Psychiatry, Institute of Psychiatry, Psychology & Neuroscience, King's College London, London, UK; and University Hospitals of Leicester NHS Trust, Leicester, UK; School of Academic Psychiatry, Institute of Psychiatry, Psychology & Neuroscience, King's College London, London, UK; and DECIPHer, School of Social Sciences, Cardiff University, UK; Royal Central School of Speech & Drama, London, UK; Group for Research in Relationships in Neurodiversity (GRRAND), Clinical, Educational & Health Psychology, Division of Psychology & Language Sciences, Faculty of Brain Sciences, University College London, London, UK; and Anna Freud National Centre for Children and Families, London, UK

**Keywords:** Autism, ADHD, adolescence, neurodiversity, patient and public involvement

## Abstract

**Background:**

There are increasing calls for neurodivergent peoples’ involvement in research into neurodevelopmental conditions. So far, however, this has tended to be achieved only through membership of external patient and public involvement (PPI) panels. The Regulating Emotions – Strengthening Adolescent Resilience (RE-STAR) programme is building a new participatory model of translational research that places young people with diagnoses of attention-deficit hyperactivity disorder (ADHD) and autism at the heart of the research team so that they can contribute to shaping and delivering its research plan.

**Aims:**

To outline the principles on which the RE-STAR participatory model is based and describe its practical implementation and benefits, especially concerning the central role of members of the Youth Researcher Panel (Y-RPers).

**Method:**

The model presented is a culmination of a 24-month process during which Y-RPers moved from advisors to co-researchers integrated within RE-STAR. It is shaped by the principles of co-intentionality. The account here was agreed following multiple iterative cycles of collaborative discussion between academic researchers, Y-RPers and other stakeholders.

**Results:**

Based on our collective reflections we offer general guidance on how to effectively integrate young people with diagnoses of ADHD and/or autism into the core of the translational research process. We also describe the specific theoretical, methodological and analytical benefits of Y-RPer involvement in RE-STAR.

**Conclusions:**

Although in its infancy, RE-STAR has demonstrated the model's potential to enrich translational science in a way that can change our understanding of the relationship between autism, ADHD and mental health. When appropriately adapted we believe the model can be applied to other types of neurodivergence and/or mental health conditions.

The rise of the neurodiversity movement has strengthened calls for neurodivergent peoples’ involvement in research into neurodevelopmental conditions such as attention-deficit hyperactivity disorder (ADHD) and autism.^1–4^ However, to date, the scale and scope of the influence of neurodivergent young people on the conduct of research that may eventually directly affect their lives has typically been limited to membership of patient and public involvement (PPI) panels. Such committees are typically constituted outside the main research team and give arms-length advice on matters such as acceptability, engagement and implementation. Such panels are now regarded as essential standard practice for clinically oriented studies. Few doubt that they have improved the conduct of clinical research by increasing the acceptability, appropriateness and ecological validity of methods.^5,6^ The dissemination of research findings and implementation of interventions have likely also become more effective because of them. While acknowledging the benefits of current practice, funders such as the National Institute for Health and Care Research (NIHR), Medical Research Council (MRC) and Wellcome Trust are now recognising the possibility of extending these PPI approaches to get patients more actively and directly involved in research programmes by taking on designated roles within research teams.

At the same time the participatory imperative at the heart of the neurodiversity movement also pushes us to go further in terms of the depth and scope of young people's involvement in research.^7–10^ In terms of scope, their influence needs to be extended to more basic translational science studies aimed at understanding the mechanisms through which neurodevelopmental variation affects young people's daily lives in ways that can be harnessed to improve intervention approaches. In terms of depth, we need to find ways to ensure that neurodivergent people's input is made across the entire scientific cycle, from shaping theory, proposing hypotheses and contributing to new methods to interpreting and disseminating findings. If this can be achieved, we believe that science will benefit both from neurodivergent people's insights into the nature of their own neurodivergences and from the different ways of thinking and working they bring to the research team. However, although in principle many accept the need to strengthen the participatory nature of translational science in this way, it remains unclear how this can be achieved in practice.

Taking up this challenge, our ongoing research programme ‘Regulating Emotions – Strengthening Adolescent Resilience’ (RE-STAR; www.kcl.ac.uk/research/re-star) is pioneering a new approach to participatory translational research, driven by this need and inspired by the neurodiversity perspective. This has involved creating and embedding a framework that enables young people with diagnoses of ADHD and/or autism to be integrated into our core research team so that their participation can evolve over time from advisors, as in traditional PPI approaches, to valued co-researchers – where they can have a greater influence on the scientific goals and conduct of RE-STAR research. The turning point in this process was the formation and development of the Youth Researcher Panel (Y-RP): a group of ten young people (so-called Y-RPers, aged 18–25 years at recruitment) all with a diagnosis of ADHD and/or autism but with little formal experience of research.

Here we outline: (a) the principles on which the RE-STAR participatory model is based; (b) its practical implementation, especially the integration of the Y-RPers; and (c) the theoretical, methodological and analytical benefits of Y-RPer involvement in RE-STAR so far.

## Method

The participatory model presented here is a culmination of a 24-month process. It is built on the concept of co-intentionality.^11^ This approach, which aspires towards a more equal and collaborative engagement between researchers and those being researched, has been at the core of the practice of socially engaged, reflexive and performative applied arts for generations. According to it, knowledge generation requires the coordinated action of all individuals across whole communities, who despite holding different types of knowledge, perspectives, interests and aptitudes, agree to work with good will and in a flexible, iterative and collaborative way towards a common goal (i.e. share the same intention). The ‘owner’ of knowledge and the source of expertise become less relevant, whereas the way that shared knowledge is applied to advance the common goals of the whole research team comes into sharper focus. This flattens the power hierarchies that have traditionally operated within science, allowing an authentic collaboration.^12^

The accounts presented below were agreed following multiple iterative cycles of collaborative discussion between academic researchers, Y-RPers and other stakeholders. During these discussions the authors of this paper each reflected on our experience of the process through which the Y-RP was formed, developed and integrated into the core of the RE-STAR team. This allowed us to describe the challenges that we have faced during the process, to specify a set of preconditions for the implementation of an effective participatory model and identify the benefits of this already seen in RE-STAR.

## Results

### Process

Through careful facilitation, staging and scaffolding using techniques from applied and social participatory research, under a clearly expressed duty of care protocol (available from the corresponding author E.J.S.S.-B.), the Y-RP members (Y-RPers) have moved from having a traditional PPI advisory role to being integrated co-researchers with involvement across all aspects of the study so far. On reflection, a number of discernible steps can be identified in this journey (see the Y-RPers’ statement, available in the supplementary material at https://dx.doi.org/10.1192/bjp.2023.151).

#### Step 1: Assembling the team

This started with a call to members of the autism and ADHD communities. We advertised, using social media, to neurodivergent-led sites/organisations/groups. There was an overwhelming response. We approached each person individually and asked why they would like to be involved in RE-STAR, and after further conversation we selected candidates and assembled as diverse a group as possible in terms of age, gender and experience, always keeping in mind their willingness and availability to work as a team.

#### Step 2: Setting the ground rules, establishing trust and developing a shared vision

We began by engaging the newly recruited Y-RPers with the research topic and explaining in detail the study aims. This was done using an approach that emphasised their value to RE-STAR and their ‘expertise by experience’. Everything was predicated on a transactional process signifying appreciation and respect. We then moved onto building the platform on which our co-intentional practice would be established. Together we drew up a duty of care protocol, outlining how the research team and Y-RPers would work together, agreeing shared expectations (available from the corresponding author E.J.S.S.-B.) and ground rules for interaction.^13^ The process of embedding the Y-RPers into research started at this stage as we invited them to provide feedback on early drafts of study documents (e.g. participant information sheets). Self-expression and sharing through performative and aesthetic practices have been especially important in building shared understandings, mutuality and trust between different members within the RE-STAR community. This provided a vehicle for the Y-RPers to explore their emotional lives. We included a range of affective approaches taken from performing arts practice, such as verbatim theatre, living library and video vignettes embedded in interview schedules, as well encouraging the Y-RPers to express themselves through modelling, animation, drawing and collage,^14–17^ a series of techniques that invited participants to find alternative ways to articulate their answers to our research questions (or even come up with different questions). Early on, the Y-RPers asked to be involved in decision-making. The research team responded by inviting them to attend leadership meetings. This was organised on a rotational basis, with one or two different Y-RPers attending different management group and steering committee meetings.

#### Step 3: Increasing confidence to co-create

Next, we worked with the Y-RPers to co-create stimuli, tasks and a topic guide to use in the data collection phase of the study. This included the Y-RPers creating short videos (vignettes) in which they talked about their emotional responses to various everyday provocations; these would then be used within the interviews with participants to stimulate discussion of their personal experiences.

#### Step 4: Developing the skills to co-research

After receiving training on the various methods being applied, a number of Y-RPers expressed keenness to be involved in the mechanics and delivery of the research itself. These individuals were then trained to co-deliver research interviews alongside the academic researchers and to undertake thematic analysis of the interview transcripts. This has led to new more nuanced understandings of the effects of ADHD and autism on young people's emotional lives. In this way Y-RPers became integrated, each to different degrees, into the research team and have been centrally involved in presenting RE-STAR's findings at scientific meetings and co-authoring papers, including this one.

It is worthy of note that when they first entered the programme Y-RPers were officially designated as a Youth Research Advisory Panel (Y-RAP). Their collective agreement to take up a fuller research role was marked by symbolically dropping the ‘A**’** from their title.

### Preconditions

Based on our collective reflections, we have identified a number of general preconditions for the effective implementation of our participatory framework.

First is a shared passion for the study and a complete commitment by all the team to the goal of improving the life chances of young people affected by autism and ADHD through the application of the translational science approach.

Second is the development of trust and confidence in each other so that everyone feels secure enough to be open about, and willing to draw on, personal experiences – this applies to both the professional researchers and the Y-RPers. This has involved conscious efforts to establish consistent and transparent communication as well as enjoyable interactive activities. Opportunities have been created to allow discussion of difficult topics relating to vested interests and power differentials openly and in good faith.

Third is a recognition that different perspectives can bring new insights to an issue and a desire to grow and learn as individuals, to reflect on and be curious about one's own assumptions and be happy to be challenged to think in new ways. In this way engagement in participatory translational research is likely to change one's ideas and perspectives on neurodivergence, mental health and the scientific process.

Fourth has been a willingness to accept the uncertainty and risk that developing a new participatory framework inevitably brings, even in a programme like RE-STAR with a well-defined structure and clear focus.

Fifth is a desire to be truly interdisciplinary, so as to respect the expertise and strengths of others in the team – whether gained through personal experience, creative research, or scientific or clinical training – irrespective of age. Ensuring that every voice in the process is heard and valued, and to demonstrate this by how people are treated.

Sixth is an understanding of the validity and value of different forms of evidence (and their limitations) – including the idea that knowledge is multifaceted and cannot be captured by words and numbers alone. Attention to affect and representation through image, sound, movement and metaphor can have intrinsic value as ways of creating and expressing knowledge.

Seventh is a facilitative sensitivity to, and scaffolding of, the diverse needs of all members of the RE-STAR community – including the provision of carefully tailored and nuanced training and mentoring for everyone, where both co-production and co-producers can thrive.

Eighth is a system of rewards and recognition to ensure that Y-RPers’ contribution is recognised in academic (authorships) and non-academic (finance) terms.

Ninth is a style of coordinated rotating leadership that harnesses the skills of all the individuals in the team and allows space for exploration of ideas and debate but maintains sufficient focus and structure to ensure progress towards milestones.

It is worthy of note that Y-RPers joined an already neurodivergent research team (six out of eight academic authors of this paper had received diagnoses of autism, ADHD or another neurodevelopmental condition) – this likely played an important role in the success of the RE-STAR model.

### Challenges

During the Y-RPers’ journey from advisors to co-researchers we have faced and overcome a number of challenges. First, the calibration of levels of support and rates of transfer of responsibility to the Y-RPers has been a core challenge, especially the need to tailor this to the needs and circumstances of each individual person. In responding to this challenge there was no alternative to investing substantial amounts of time to understand each Y-RPer's situation, interests and motivations and to pace the process so that all progressed at their own rate towards a common goal. Second, it has sometimes proven challenging for the professional researchers on RE-STAR to communicate technical scientific terms and concepts or participatory research jargon in a way that is understandable to the Y-RPers. Y-RPers have been good at saying when ideas are difficult to follow or are unclear and asking for explanations. Third, all had to be willing to work outside office hours. Fourth, the Y-RPers are all busy people and the extent to which they have time get involved in RE-STAR varies considerably, depending on their availability. It is essential that Y-RPers feel comfortable with the amount time they contribute to the project, engage in activities meaningful to them and receive appropriate remuneration for it. Fifth, it proved difficult to attract applications from young people with diagnoses of ADHD and autism from ethnic minority groups and the initial group lacked diversity of this kind. However, we have now recruited a second set of Y-RPers with greater ethnic variety.

Beyond these challenges, RE-STAR looks to celebrate Y-RPers’ contribution in various ways.

### Contributions

Now in their third year in RE-STAR, the Y-RPers have made a substantive contribution to the RE-STAR programme that has driven it in new and exciting directions. For instance, within the first part of the programme, an interview study exploring the emotional lives of young people with a diagnosis of ADHD and/or autism, the Y-RP has benefited RE-STAR in the following ways.

- Its members have influenced the development of ideas on the link between neurodivergence and mental health and ill-health. In particular, they have highlighted the need for theories to more fully incorporate the role of context in determining ADHD- and autism-related emotional experiences in a way that is shaping our experimental hypotheses.
- They changed the way outcomes are measured and processes are captured. They have enhanced methodological creativity through the co-design of novel, experience-sensitive ways of gathering information during interviews, through art, poetry and prose. These have facilitated access to understandings that some participants have found difficult to articulate verbally.
- They have promoted new ways of collecting data by co-interviewing alongside academic researchers. This has changed the dynamic of the data collection process, allowing us to access different perspectives.
- They have allowed more valid interpretation of data by participating in analysis to identify themes of importance from their perspective, which have provided a platform for the experimental studies to follow.
- They have driven creative knowledge exchange. They developed and delivered a multimedia public engagement event that widened the conversation to other stakeholders in this inquiry, such as parents and carers, and foregrounded applied arts as research methods. The event, ‘My Emotions and Me: A Journey Behind the Mask’, was presented at the 2022 Being Human Festival – the UK's national festival for the humanities. It brought together Y-RPers, artists and scientists to explore how partnership, collaboration and co-research can enhance current understanding of neurodivergence and mental health. Y-RPers routinely share the conference stage as equal contributors (e.g. EUNETHYDIS 2022 and ITAKOM 2023) – where talk about science and their own experience are interwoven.

## Discussion

We have set out in this paper a co-intentional approach to participatory translational science that is aligned with contemporary neurodiversity perspectives. As detailed above, it has so far proved feasible to implement and has added considerable value to the early stages of the RE-STAR programme. It is our shared hope that by adopting this approach of integrating adolescents with diagnoses of ADHD and autism into the scientific process, RE-STAR will lead to fundamental changes in our understanding of the role of emotional experience in the mental health and ill-health of neurodivergent young people that can be translated into more effective interventions. As we move forward in RE-STAR, we are working together to ensure the findings from the early work packages are used to inform subsequent work packages. In particular, it is fascinating to see how Y-RPers are continuing to shape the content and practice of more laboratory-based experimental and longitudinal research as we move through the translational cycle.

The plan at the outset was to supplement the original Y-RPers, once established in their roles, with younger people – closer to the age of RE-STAR participants, whom they would guide and mentor. In fulfilment of this we have appointed seven young people between the ages of 11 and 16 years. These young people are now being integrated into the team. In this case the parents are playing a greater role supporting their children. For example, they joined the initial induction session, and in subsequent sessions we set up parallel break-out rooms for parents to share experiences.

### Research implications

One obvious question is whether the RE-STAR framework can be generalised to individuals with other forms of neurodivergence, say dyslexia or dyspraxia, or those with subclinical presentations, or those with mental health conditions such an anxiety or depression. We believe the general philosophical framework (co-intentionality) and the processes through which the Y-RP has been established and developed are likely to bring value to participatory research with other groups of neurodivergent individuals. Applying these general principles and processes will ensure that specific models of participation will be appropriately tailored to the needs of the specific young people on which a study is focused – in the way that the RE-STAR model has been developed out of the interactions between neurodivergent young people and the wider RE-STAR team. In the future, it may be efficient for studies of neurodevelopmental and mental health conditions using participatory approaches to build on the RE-STAR framework and procedures as a jumping off point for collaborations with different groups of neurodivergent individuals.

## Supporting information

Sonuga-Barke et al. supplementary material Sonuga-Barke et al. supplementary material

## Data Availability

Data availability is not applicable to this article as no new data were created or analysed in this work.
