# How can we move beyond current *Patient and Public Involvement* models to enrich translational science*?* A framework for integrating autistic people and those with ADHD into research practice

**DOI:** 10.1192/bjp.2023.151

**Published:** 2024-02-16

**Authors:** Edmund J S Sonuga-Barke, Susie Chandler, Steve Lukito, Myrofora Kakoulidou, Graham Moore, Niki Cooper, Maciej Matejko, Isabel Jackson, Beta Balwani, Tiegan Boyens, Dorian Poulton, Luke Harvey-Nguyen, Sylvan Baker, Georgia Pavlopoulou, The RE-STAR team, Edmund Sonuga-Barke, Susie Chandler, Andrea Danese, Johnny Downs, Myrofora Kakoulidou, Steve Lukito, Angus Roberts, Emily Simonoff, Daniel Stahl, Georgia Pavlopoulou, Jane Hurry, Sylvan Baker, Graham Moore, Dennis Ougrin, Ned Redmore, Niki Cooper, Amy Locke, Youth Researcher Panel members, Beta Balwani, Tiegan Boyens, Zoe Glen, Luke Harvey-Nguyen, Issy Jackson, Elisa Ly, Elizabeth Macauley, Maciej Matejko, Dorian Poulton, Archie Wilson

**Affiliations:** King’s College London; Anna Freud Centre and UCL; University College London; Royal Central School of Speech and Drama; Cardiff University; Queen Mary University of London; Autistica; Place2Be; ADHD Foundation; 1 School of Academic Psychiatry, Institute of Psychiatry, Psychology & Neuroscience, King’s College London, UK; 2 Royal Central School of Speech & Drama, London, UK; 3 Group for Research in Relationships in Neurodiversity (GRRAND), Clinical, Educational & Health Psychology, Division of Psychology & Language Sciences, Faculty of Brain Sciences, University College London, UK; 4 University Hospitals of Leicester NHS Trust, UK; 5 Anna Freud National Centre for Children and Families, London, UK; 6 DECIPHer, School of Social Sciences, Cardiff University, UK; 7 Niki Cooper, Place2Be. UK

## Abstract

With its battle cry of “nothing about us without us”, the neurodiversity movement has strengthened neurodivergent peoples’, such as those with ADHD, autism, dyslexia and dyspraxia, involvement in research. So far, however, this has tended to be limited to more clinically oriented studies, as opposed to basic translational science, and then only to membership of advisory panels such as Patient and Public Involvement (PPI) committees. These typically sit outside the main research team to give arms-length advice on matters of acceptability, engagement, and implementation.

The *Regulating Emotions – Strengthening Adolescent Resilience* (RE-STAR) programme is developing new science-driven ways to reduce the depression risk in Autistic Adolescents and those with ADHD (AAA). At the same time, RE-STAR is developing a new participatory model of AAA translational research. Here, we outline the principles on which this approach is based and its practical implementation, especially the central role of the Youth Researcher Panel (Y-RP), mapping out the panel members’ journey from advisor to co-researcher. We reflect on the feasibility of this participatory approach, implementation challenges faced, and insights gained. We describe the benefits it has brought when implemented with care and attention.

Integrating AAA into the research process in this way has the potential to enrich translational neuroscience, leading to fundamental changes in our understanding of the relationship between autism, ADHD and mental health, and increasing the chance of more effective interventions. We also recommend that the model, when appropriately adapted, can be applied to other types of neurodivergence and/or mental health conditions.

## 1) Neurodivergent peoples’ participation in research: The need to go further and deeper

The rise of the neurodiversity movement has strengthened the participatory imperative in research into neurodivergences such as ADHD, autism, dyslexia and dyspraxia. However, to date, the scale and scope of the influence of neurodivergent young people on the conduct of research that directly affects their lives has typically been limited to membership of *Patient & Public Involvement* (PPI) committees. Such committees are so far typically constituted *outside* the main research team to give arms-length advice on matters of acceptability, engagement, and implementation. This is now essential standard practice for clinically oriented studies and few doubt that it has improved the conduct of clinical research by increasing the acceptability, appropriateness and ecological validity of methods ^[5]^. The dissemination of research findings and intervention implementation have probably also improved because of it. While acknowledging the benefits of current practice, funders such as the NIHR, MRC and the Wellcome Trust are now recognising the possibility of extending these PPI approaches to get patients more actively involved in research programmes by taking on designated roles *within* research teams.

At the same time the participatory imperative at the heart of the neurodiversity movement, with its battle cry of “nothing about us without us”, also pushes us to go further in terms of the *depth* and *scope* of young people’s involvement in research ^[7]^. In terms of *scope*, their influence needs to be extended to more basic translational science studies aimed at understanding the mechanisms through which neuro-developmental variation impacts young people’s daily lives, in ways that can be harnessed to improve intervention approaches. In terms of *depth*, we need to find ways to ensure neurodivergent people’s input is made across the entire scientific cycle from shaping theory, proposing hypotheses, contributing to new methods, and interpreting and disseminating findings. If this can be achieved, we believe that science will benefit both from neurodivergent people’s insights into the nature of their own neurodivergences as well as because of the different ways of thinking and working they bring to the research team. However, while, in principle, the need to strengthen the participatory nature of translational science in this way is generally accepted, it remains unclear how this can be achieved in practice.

Taking up this challenge, our ongoing *Regulating Emotions – Strengthening Adolescent Resilience* (RE-STAR) research programme on depression in Autistic Adolescents and those with ADHD (AAA), is pioneering a new approach to participatory translational research driven by this need and inspired by the neurodiversity perspective. This has involved creating and embedding a framework that enables AAA to be integrated into our core research team – with their participation having evolved over time from being advisors, as in traditional PPI approaches, to valued co-researchers – where they can have a greater influence on the scientific goals and conduct of RE-STAR research.

## 2) Integrating AAA into the core research team: The Youth Researcher Panel (Y-RP) and its impact

The turning point in this process was the formation and development of the Youth Researcher Panel (Y-RP). Initially, the Y-RP comprised a group of ten young people (aged 18-25 years at recruitment) all with a diagnosis of ADHD and/or autism but with little formal experience of research. It is worthy of note that they joined a research team that included researchers who have been formally identified as having either ADHD or autism themselves (including six out of eight named academic authors of this article).Through careful facilitation, staging and scaffolding using techniques from applied and social participatory research, under a clearly expressed duty of care protocol, the Y-RP members (Y-RPers) have moved from having a traditional PPI advisory role to fully integrated researcher participation with involvement across all aspects of the study so far.

Although just in their second year in RE-STAR, the Y-RP co-researchers have made a substantive contribution to the RE-STAR programme that has shaped it in new and exciting directions. Within the first part of the programme, an interview study exploring the emotional lives of AAA, the Y-RP has benefited RE-STAR in the following ways. They have:

(i) influenced the development of ideas on the link between neurodivergence and mental health. In particular, they have highlighted the need for theories to incorporate the role of context in determining AAA emotional experiences in a way that is shaping our experimental hypotheses.
(ii) changed the way outcomes are measured and process are captured. They have enhanced methodological creativity through the co-design of novel, experience-sensitive ways of gathering information during interviews, through art, poetry, and prose. These have facilitated access to understandings that some participants have found difficult to articulate verbally.
(iii) promoted new ways to collect data by co-interviewing AAA alongside academic researchers. This has changed the dynamic of the data collection process allowing us to access different perspectives.
(iv) allowed more valid interpretation of data by participating in analysis to identify themes of importance from their perspective which have provided a platform for the experimental studies to follow.
(v) driven creative knowledge exchange. They developed and delivered a multimedia public engagement event that widened the conversation to other stakeholders in this inquiry, such as parents and carers and foregrounded applied arts as research methods – *My emotions and me: A journey behind the mask* – at the 2022 Being Human Festival – the UK’s national festival for the humanities. The event brought together Y-RPers, artists and scientists to explore how partnership, collaboration, and co-research can enhance current understanding of neurodivergence and mental health. Y-RPers routinely share the conference stage as equal contributors (Eunethydis 2022 and ITAKOM 2023) – where talk about science and their own experience are interwoven.

## 3) Key principles and essential preconditions

Essential to creating the conditions allowing this shift from arms-length involvement to the positioning of Y-RPers at the heart of the research team is the concept of co-intentionality ^[11]^. This approach which aspires towards a more equal and collaborative engagement between researchers and participants, has been at the core of socially engaged, reflexive and performative arts-practice research for generations. According to it, knowledge generation requires the co-ordinated action of all individuals within a community, who despite holding different types of knowledge, perspectives, interests and aptitudes agree to work, with good will and in a flexible, iterative and collaborative way towards a common goal (i.e., share the same intention). The ‘owner’ of knowledge and the source of expertise become less relevant, while the way that shared knowledge is applied to advance the common goals of the whole research team, comes into deeper focus. This flattens traditional power hierarchies, that have traditionally operated within science, allowing a more authentic collaboration rather than positioning lived experience as purely advisory. Based on our shared experience, there are a number of pre-conditions for the effective implementation of this framework.

First is a shared passion for the study and a complete commitment by all to the goal of improving the life chances of AAA through the translational science approach.

Second is the development of trust and confidence in each other so that everyone feels secure enough to be open about, and willing to draw on, personal experiences – this applies to both the professional researchers and the Y-RPers. This has involved conscious efforts to establish consistent and transparent communication as well as fun interactive activities. Opportunities have been created to allow discussion of difficult topics relating to vested interests and power differentials openly and in good faith.

Third is an acceptance that different perspectives can bring insight to an issue and a desire to grow and learn as individuals, to reflect on and be curious about one’s own assumptions and be happy to be challenged to think in new ways. In this way engagement in the sort of research is likely to change one’s ideas and perspectives on neurodiversity and mental health and the scientific process.

Fourth has been a willingness to accept the uncertainty and risk that developing a new participatory framework inevitably brings even in a programme like RE-STAR with a well-defined structure and clear focus.

Fifth is to be truly interdisciplinary, so as to respect the expertise and strengths of others in the community – whether gained through personal experience, creative research, or scientific or clinical training – irrespective of age. Ensuring that every voice in the process is heard and valued, and to demonstrate this in the way people are treated.

Sixth is an understanding of the validity and value of different forms of evidence (and their limitations) – including the idea that knowledge is multifaceted and cannot be captured by words and numbers alone. That attention to affect and representation through image, sound, movement and metaphor can have intrinsic value as ways of creating and expressing knowledge.

Seventh is a facilitative sensitivity to, and scaffolding of, the diverse needs of all members of the RE-STAR community – including the provision of carefully tailored and nuanced training and mentoring for everyone where both co-production and coproducers can thrive.

Eighth is a system of rewards and recognition to ensure Y-RPers’ contribution is recognised in academic (authorships) and non-academic (finance) terms.

Ninth is a style of coordinated rotating leadership that harnesses the skills of all the individuals of the team and allows space for exploration of ideas and debate but maintains sufficient focus and structure to ensure progress towards milestones

## 4) The process of moving from advisor to co-researcher

In section 2 we outlined some of the positive impacts of the involvement of the Y-RPers here we focus on the process by which these benefits were achieved and some of the challenges that have been overcome along the way. On reflection a number of discernible steps can be identified in the Y-RPers’ journey from advisor to co-researcher (please see the Y-RPers statement about this here).

### Step 1: Assembling the team

This started with a call to members of the autistic and ADHD communities. We advertised through social media to neurodivergent-led sites/organisations/groups. There was an overwhelming response. We approached each person individually and asked why they would like to be involved in RE-STAR and after further conversation, we selected candidates and assembled as diverse a group as possible in terms of age, sex/gender, and experience, always keeping in mind their willingness and availability to work as a team.

### Step 2: Setting the ground-rules, establishing trust, and developing a shared vision

We began by engaging the newly recruited Y-RPers with the research topic and explaining in detail the study aims. This was done with an approach that viewed them and their expertise by experience on the same level as that garnered in other ways. Everything was predicated on an adult-to-adult transaction process signifying appreciation and respect. We then moved onto building the platform on which our co-intentional practice would be established. Together we drew up a Duty of Care protocol, outlining how the research team and Y-RPers would work together, agreeing shared expectations (available online here) and ground-rules for interaction. The process of embedding the Y-RPers into research started at this stage as we invited the Y-RP to provide feedback on early drafts of study documents (e.g., participant information sheets). Self-expression and sharing through performative and aesthetic practices were especially important in building shared understandings, mutuality, and trust between different members within the RE-STAR community. They have provided a vehicle for self-exploration and understanding of emotion by the Y-RPers. We included a range of affective approaches taken from performance such as verbatim theatre, living library and video vignettes embedded in interview schedules, as well encouraging them to express themselves through modelling, animation, drawing and collage^[15, 16]^; a series of techniques that might invite participants to find alternative ways to articulate their answers to our research questions (or even come up with different questions). Early on the Y-RPers asked to be involved in decision making. The research team responded by inviting them to attend leadership meetings. This was organised on a rotational basis with one or two different Y-RPers attending different management group and steering committee meetings.

### Step 3: Increasing confidence to co-create

Next, we worked with the Y-RPers to cocreate stimuli, tasks and a topic guide to use in the data collection phase of the study. This included the Y-RPers creating short videos (vignettes) in which they talked about their emotional responses to various everyday triggers; these would then be used within the interviews with young people to stimulate discussion around their own experiences.

### Step 4: Developing the skills to co-research

After receiving training on the various methods being applied a number of Y-RPers expressed keenness to be involved in the mechanics and delivery of the research itself. They were then trained to codeliver research interviews alongside the academic researchers and to undertake thematic analysis of the interview transcripts. This has led to new nuanced understandings of the emotional lives of AAA. In this way the Y-RPers are now integrated, to different degrees, into the research team and as such have been centrally involved in presenting RE-STAR’s findings at scientific meetings and co-authoring papers, including this one.

During the YRPers’ journey from advisors to co-researchers we have faced a number of challenges to ensure our vision of co-intentionality. First, it proved challenging to attract applications from AAA’s from ethnic minority groups and the initial group lacked diversity of this kind. However, we have recruited a second set of YRPers with greater ethnic variation. Second, we were challenged to calibrate levels of support and rates of transfer of responsibility to the Y-RPers and to tailor this to the needs and circumstances of each individual member. In responding to this challenge there was no alternative to investing time to understand each Y-RPers situation, interests and motivations and to pace the process so that all progressed at their own rate towards a common goal. Third, it has sometimes proven challenging for the professional researchers on RE-STAR to communicate technical scientific terms and concepts or participatory research jargon in a way is legible to the Y-RPers. Y-RPers have been great at saying when this is the case and asking for explanations. Fourth, all had to be willing to work outside office hours. Fifth, the Y-RPers are all busy people and the extent to which they have time get involved RE-STAR varies considerably, depending on their availability. It is essential that Y-RPers feel comfortable with the amount time they contributed into the project, engage in activities meaningful to them and receive the appropriate reimbursement for it. Beyond this, RE-STAR looks to celebrate Y-RPers’ contribution in different ways. Y-RPers started by taking on the traditional advisory role and were designated as a *Youth Research **Advisory** Panel* (Y-R**A**P) in their initial involvement with RE-STAR. While anticipated by members of RE-STAR, the journey of the Y-RPers from being advisors to co-researchers, involves many challenges to overcome. Y-Rpers’ collective agreement to take up a fuller research role with the tailored support and training by the broader RE-STAR team, was marked by symbolically dropping the ***‘A’*** from their title.

## 5) Next Steps - Building on progress to ensure continued growth

We have already made substantial progress in creating and implementing a new participatory framework for translational research that has made it possible for the insights, experiences, attitudes, and skills of AAA to directly and substantially impact the research process. As we move forward in RE-STAR, we are working together to ensure the findings from the first part of RE-STAR inform the development of hypotheses, methods and delivery of the subsequent RE-STAR studies. In particular, it is fascinating to see how Y-RPers are continuing to shape the content and practice, given that these next steps will include experimental and longitudinal research, as we move through the translational cycle.

The plan at the outset was to supplement the original Y-RPers, once established in their roles, with younger people – closer to the age of RE-STAR participants, whom they would guide and mentor. In fulfilment of this we have appointed seven young people between the ages of 11 and 16 years. They are now being integrated into the team. In this case the parents are playing a greater role supporting their children. For example, they joined the initial induction session, and in subsequent sessions we set up parallel break-out rooms for parents to share experiences.

*In summary*, we have set out a co-intentional approach to participatory translational science that we believe is consonant with contemporary neurodiversity perspectives. As detailed above, this has so far proved feasible to implement and has added considerable value to the early stages of the RE-STAR project. Looking forward, it is our shared hope that by adopting this approach of integrating AAA in the scientific process, RE-STAR will lead to fundamental changes in our understanding the role of their emotional experience in their mental health which can be translated into more effective interventions.

One obvious question is - *Can the RE-STAR framework be generalised to individuals with other forms of neurodivergence, say dyslexia or dyspraxia, or those with sub-clinical presentations, or those with mental health conditions such an anxiety or depression?* We believe the general philosophical framework (co-intentionality) and the processes through which the Y-RP has been established and developed are likely to bring value to participatory research with other groups of neurodivergent individuals. Applying these general principles and processes will ensure that specific models of engagement with research will be appropriately tailored to the needs of the specific young people on which a study is focused – in the way that the RE-STAR model has been developed out of the interactions between autistic young people and those with ADHD and the wider RE-STAR team. In the future, it may be efficient for studies of neurodevelopmental and mental health conditions using participatory approaches to build upon the RE-STAR framework and procedures as a jumping off point for collaborations with different groups of neurodivergent individuals.

## Statement from the Youth Researcher Panel (Y-RP)

The role of Y-RP in RE-STAR has expanded from advisors to co-researchers. As the academic team have paid heed to our contributions and suggestions, we feel we have a real influence on the project. We have expressed an interest in learning about and getting involved in different aspects of the research process in ways that have often exceeded the academic researchers’ initial expectations. The academic team has been receptive to our suggestions, and our involvement is increasing as the project develops. We believe that our efforts help guide the project to be more accessible for its research participants and relevant to the well-being of neurodivergent people. The unique value the Y-RP brings to the project is the members’ lived experience of neurodivergence. This enables us to reflect on issues studied in the project in a sensitive and thorough manner and identify things that would have otherwise gone unnoticed. More broadly, we hope our involvement will help the scientific community see that this type of collaboration can be successful. We hope that RE-STAR will encourage more researchers to meaningfully engage neurodivergent people in projects about neurodiversity, as well as value the findings and opinions of neurodivergent scholars and commenters. We believe it is a long overdue step in the right direction after decades of research that has too often failed to fairly represent the people it studied or ultimately benefit them. Not only does this approach empower neurodivergent people, but we maintain that incorporating the neurodivergent perspective makes for more credible research on neurodiversity.
