# Supplementary material for: Participatory translational science of neurodivergence: model for attention-deficit/hyperactivity disorder and autism research
Source: Br J Psychiatry. 2024 Apr;224(4):127–31. doi: 10.1192/bjp.2023.151 (PMC10933558; doi:10.1192/bjp.2023.151)
Supplement: Sonuga-Barke et al. supplementary material [file S0007125023001514sup001.pdf]

Moving beyond current Patient and Public Involvement (PPI) models of participation to advance the translational science of attention-deficit/hyperactivity disorder and autism: The RE-STAR framework.

Statement from the Youth Researcher Panel (Y-RP)

The role of Y-RP in RE-STAR has expanded from advisors to co-researchers. As the academic team have paid heed to our contributions and suggestions, we feel we have a real influence on the project. We have expressed an interest in learning about and getting involved in different aspects of the research process in ways that have often exceeded the academic researchers' initial expectations. The academic team has been receptive to our suggestions, and our involvement is increasing as the project develops. We believe that our efforts help guide the project to be more accessible for its research participants and relevant to the well-being of neurodivergent people. The unique value the Y-RP brings to the project is the members' lived experience of neurodivergence. This enables us to reflect on issues studied in the project in a sensitive and thorough manner and identify things that would have otherwise gone unnoticed. More broadly, we hope our involvement will help the scientific community see that this type of collaboration can be successful. We hope that RE-STAR will encourage more researchers to meaningfully engage neurodivergent people in projects about neurodiversity, as well as value the findings and opinions of neurodivergent scholars and commenters. We believe it is a long overdue step in the right direction after decades of research that has too often failed to fairly represent the people it studied or ultimately benefit them. Not only does this approach empower neurodivergent people, but we maintain that incorporating the neurodivergent perspective makes for more credible research on neurodiversity.
